# Variability in Physical Activity Assessed with Accelerometer Is an Independent Predictor of Mortality in CHF Patients

**DOI:** 10.1371/journal.pone.0153036

**Published:** 2016-04-07

**Authors:** Michael Melin, Inger Hagerman, Adrian Gonon, Thomas Gustafsson, Eric Rullman

**Affiliations:** 1 Department of Cardiology, Karolinska Institutet, Karolinska University Hospital, Huddinge, Stockholm, Sweden; 2 Division of Clinical Physiology, Department Laboratory Medicine, Karolinska Institutet, Karolinska University Hospital, Huddinge, Stockholm, Sweden; University of Naples Federico II, ITALY

## Abstract

**Aims:**

Patients with heart failure often display a distinct pattern of walking characterized by short step-length and frequent short pauses. In the current study we sought to explore if qualitative aspects of movement have any additive value to established factors to predict all-cause mortality in patients with advanced heart failure.

**Methods and results:**

60 patients with advanced heart failure (NYHA III, peak VO2 <20 ml/kg and LVEF <35%) underwent symptom-limited CPX, echocardiography and routine chemistry. Physical activity was assessed using an accelerometer worn attached to the waist during waking hours for 7 consecutive days. The heart-failure survival score (HFSS) was calculated for each patient. All accelerometer-derived variables were analyzed with regard to all-cause mortality and added to a baseline model utilizing HFSS scores. HFSS score was significantly associated with the incidence of death (P<0.001; c-index 0.71; CI, 0.67–0.73). The addition of peak skewness to the HFSS model significantly improved the predictive ability with an increase in c-index to 0.74 (CI, 0.69–0.78), likelihood ratio P<0.02, establishing skewness as a predictor of increased event rates when accounting for baseline risk.

**Conclusion:**

The feature skewness, a measure of asymmetry in the intensity level of periods of high physical activity, was identified to be predictive of all-cause mortality independent of the established prognostic model–HFSS and peak VO2. The findings from the present study emphasize the use of accelerometer analysis in clinical practice to make more accurate prognoses in addition to extract features of physical activity relevant to functional classification.

## Introduction

Heart failure (HF) is a common disease with an estimated prevalence of about 2% in the population as a whole and almost 10% among the elderly [1,2]. Despite significant improvements in the treatment of chronic HF, it remains one of the deadliest diseases known, with one-year mortality ranging from 5% up to 40% depending on severity of the disease. For patients with severe heart failure the ultimate treatment option remains transplantation, which improves the prognosis in both the short and the long term [3–5]. Left ventricular assist devices (LVAD) were recently introduced as a promising option for patients with a serious prognosis [6]. As these procedures are associated with both a considerable cost and risk, it is crucial to select patients who would have the greatest benefit [3,4,6,7]. Consequently, an important challenge is to identify individuals with a high risk of death before the severity of the disease makes invasive measures impossible. As a result, a large number of biomarkers and clinical prediction models have been introduced with the aim of identifying patients at risk of early death [8]. Physical capacity, measured as peak VO2, has repeatedly been shown to be one of the strongest predictors of mortality in heart failure [5,9–11]. Most algorithms use however several variables to estimate the risk of mortality or adverse events [12]. The heart-failure survival score (HFSS) prediction model which combine peak VO2 with selected clinical variables is perhaps the most established prediction model in heart failure to date [13,14].

Daily physical activity (PA) is well characterized as being linked to several aspects of both health and disease in the general population [15–17]. PA is recommended as a beneficial adjunctive treatment in symptomatic HF patients with reduced left ventricular ejection fraction, however the effect on mortality remains unclear [18,19]. Assessment of PA using accelerometers has become increasingly popular as the size and cost of these monitors have decreased [16]. In most studies these measurements serve the purpose of estimating total energy expenditure and/or assessing the amount of time each participant is active. In patients with HF, it was demonstrated that individuals with higher daily PA belonged to a higher functional class [20]. Further, a very innovative approach was carried out recently in a post-hoc analysis of the SENSE-HF and DOT-HF studies: By using records from the accelerometers integrated in HF devices (CRTPs and ICDs), the total time spent being physically active (regardless of intensity) versus time spent being physically inactive could predict adverse clinical events in patients with moderate to severe HF [21]. However, a limitation with this approach is the low temporal resolution. In contrast to HF devices, external accelerometers store information with a higher temporal resolution–often 1 minute–compared with pacemaker devices, which only store daily summaries. The higher temporal resolution enables calculations of numerous other variables related to the individual daily activity. Still, regardless of the device used, estimating PA using accelerometers is tedious and technically challenging when performed on inactive subjects. Furthermore supervised intervention studies indicate that time spent being physically active is tightly linked with physical capacity and peak VO2 [19,22], which may therefore undermine the possibility to further increase the predictive value above what obtained with measurements of physical capacity. Thereby, to be able to identify variables related to disease progress without any relation to physical capacity may be more successful to add substantial predictive value in the various clinically utilized algorithms. A widely recognized hallmark of severe HF is a changed pattern of walking, characterized by short step length and frequent short pauses [23]. It is plausible that PA measured by accelerometers would identify such pattern as a high degree of variability due to the frequent start and stops and the inability to maintain constant speed of motion. In the present study we tested whether a high degree of variability in PA could be identified and characterized through an analysis of accelerometer data and if the degree of variability would contribute an additive value above that of physical capacity and other established risk factors, in a prognostic model.

## Material and Methods

### Patient population and study design

Patients were prospectively enrolled at the outpatient clinic at Karolinska University Hospital. Patients with moderate to severe and stable chronic HF defined as functional class NYHA III with no acute hospital admission within the last 8 weeks were eligible for inclusion. NYHA III was defined as a self-reported maximum continuous walking distance of no more than 200 m with dyspnea as a limiting factor. During scheduled visits to the attending cardiologist patients fulfilling the inclusion criteria were invited to participate. 60 patients were recruited between May 2009 and June 2013. This is an explorative study and the variables were not predefined.

All patients signed a written informed consent form. The study was approved by the Regional Ethical Review Board in Stockholm, Sweden (nr 2007/1410-31/3). The study has been carried out in accordance with The Code of Ethics of the World Medical Association (Declaration of Helsinki). All patients underwent routine echocardiography, blood sampling (summarized in Table 1), symptom-limited cardiopulmonary exercise test (CPX) with measurement of peak VO2. CPX on a treadmill consisted of maximum symptom-limited bicycle or treadmill exercise (1 m/s with a stepwise increase in the angle of grade /min) and continuous assessment of gas-exchange data (Vmax, SensorMedics, Anaheim, CA, USA). In all cases the exercise was terminated due to volitional exhaustion and/or the patient’s inability to maintain the speed of 1 m/s despite strong verbal encouragement. Daily activity was assessed by an accelerometer (GT3X; Actigraph, Pensacola, FL, USA), which was mailed to all patients within 6 weeks of the baseline characterization. The patients were instructed to attach the accelerometer to their waist belt upon rising in the morning and to remove it only for showering, bathing and sleeping. The monitors were set to begin collecting data one day before the delivery date, as estimated by the postal service, and to continue recording data until they were downloaded. The patients were asked to return the monitor by mail using a prepaid return envelope after having worn it for 7 days. 12–56 months following baseline data collection data on mortality and cause of death were obtained from the Swedish national cause-of-death registry.

**Table 1:** Baseline clinical characteristics and clinical characteristics of patients with high vs. low 3 h skewness.

		Peak skewness		
Variable	All patients	Above Median	Below Median	p-value
5-year all-cause mortality (%)	41.1	57.1	25.0	0.06
Age (years)	70.3	70.5	70.1	0.80
Ejection fraction (%)	23.8	25.0	24.9	0.98
Heart rate (BPM)	74.9	75.7	74.2	0.73
Mean arterial pressure (mm Hg)	90.2	89.3	91.1	0.68
Peak VO2 (ml/kg)	10.2	10.2	10.3	0.78
HFSS (a.u)	8.5	8.4	8.5	0.77
Sodium (mmol/l)	140.1	139.9	140.3	0.76
Hemoglobin (g/l)	141.5	139.3	143.7	0.37
Female (%)	23.2	21.4	25.0	0.78
Body mass index (kg/m2)	28.7	28.6	28.8	0.89
Glomerular filtration rate (ml/min)	65.9	63.1	68.7	0.45
NT-proBNP (ng/l)	3727	3969	3485	0.70
Implantable cardioverter defibrillator (%)	57.1	53.6	60.7	0.72
ACEi/ARB-blockers (%)	94.6	92.9	96.4	0.89
Beta-blockers (%)	96.5	100.0	92.9	0.79
Atrial fibrillation (%)	60.7	50.0	71.4	0.30
Diabetes (%)	42.9	46.4	39.3	0.68
Kidney failure (%)	28.6	25.0	32.1	0.62
Chronic Obstructive Pulmonary Disease (%)	16.1	25.0	7.1	0.10
Ischemic heart disease (%)	57.1	57.1	57.1	1.00
QRS-duration >120 ms (%)	33.9	32.1	35.7	0.82

All patients had advanced heart failure receiving adequate medical treatment. Established risk-factors were equally distributed between the groups. Continuous variables were tested using t-test and frequencies using chi-square test.

BPM: Beats per minute

HFSS: Heart Failure Survival Score

eGFR: Estimated glomerular filtration rate

COPD: chronic obstructive pulmonary disease

ACEi: angioconverting enzyme inhibitor

ARB: angiotensin receptor blocker

### Accelerometer analysis

Raw data collected by the accelerometer were integrated into 60-second epochs using ActiLife software using the normal filter option and expressed as counts per minutes (cpm). Wear time was estimated using the algorithm described by Troiano et al [24]. Non-wear time was defined as 60 consecutive minutes of 0 cpm, with allowance for 1–2 minutes of 0–99 cpm during this time. Patients with an estimated wear-time of <3 days were eliminated from further analysis (n = 3). To overcome the limitations that come with the use of heuristic features based on a healthy population and the uncertainty regarding wear-time estimation we also employed an additional, different approach: first episodes of more or less continuous physical activity were identified by applying a rolling mean to the raw data (epochs per minute on the vertical axis) and 1-, 3- and 12-hour period with the highest mean activity over the recorded 7-day period were collected. In these three time periods an average activity was measured (mean, median, max, min). The variables used for pattern recognition (measures of variance) in accelerometers (IQR, skewness and kurtosis) were calculated. A high degree of skewness indicates that the activity level is high during a particular time in the recorded period, whereas a constant level of activity renders a low degree of skewness. Kurtosis, on the other hand, is a measure of “peakedness”; therefore, a constant level of activity also implies a low degree of kurtosis. However, kurtosis becomes high when there are many short burst of activity over the period of interest. In addition, the following summary accelerometric metrics were estimated: 1) total number of minutes the monitor was worn; 2) sedentary time (vertical axis cpm <100); 3) light activity (vertical axis cpm between 100 and 1951) time; 4) moderate vigorous physical activity (vertical axis cpm greater than 1952) time, where 1952 counts per minute corresponds to walking at 4 km/h [25].

All data analysis was carried out on the R 3.0.1 platform using the “Physical Activity” and “e1071” packages.

### Score calculation and statistical analysis

HFSS was calculated by summarizing the beta-coefficients of peak VO2, LVEF, resting heart rate, serum sodium, ischemic etiology (categorical) and LBBB/RBBB (categorical) for each patient in accordance with Aaronson et al.:

ischemic etiology x 0.6931 + resting heart rate x 0.0216 + LVEF(%) x -0.0464 + mean arterial blood pressure x -0.0255 + intraventricular conduction delay x 0.6083 + peak VO2 (ml/min/kg) x -0.0546 + serum Na (mmol/L) x -0.047 (13). Cox proportional hazards regression was used to analyze the relationship between mortality and accelerometer data, all of which were analyzed as continuous variables. The hazard ratio (HR) and its associated 95% confidence interval (CI) were reported for each variable. The incidence of end points is illustrated using Kaplan–Meier/Cox-regression graphs with patients being divided into 2 equal-sized groups based on values below or above median.

The study was explorative and data-driven in the sense that the algorithms, calculations and resulting variables were not predefined but was established once the data was collected. Therefore, no *a priori* power-calculation of the cox-regressions was performed. However, based on the empirical value distribution, power for the cox-regression was calculated according to Hsieh et al: Log-hazard ratio per unit increase in skewness = 1.4 (coefficient of the regression, see Table 1). Standard deviation of skewness in the cohort was = 1.89. With an alpha (i.e p-value) of 0.05 this gives a power of 0.79 in univariate cox-regression analysis [26]. All analyses were done using R (version 3.0.1; the R project for statistical computing; www.r-project.org), along with the “survival” and “pec” and “powerSurvEpi” packages. Biplot was generated using the “bpca” package. The results were reported using mean and standard deviations for continuous variables and counts and percentages for categorical variables.

All data are to be found in S1 File.

## Results

**Fig 1:**
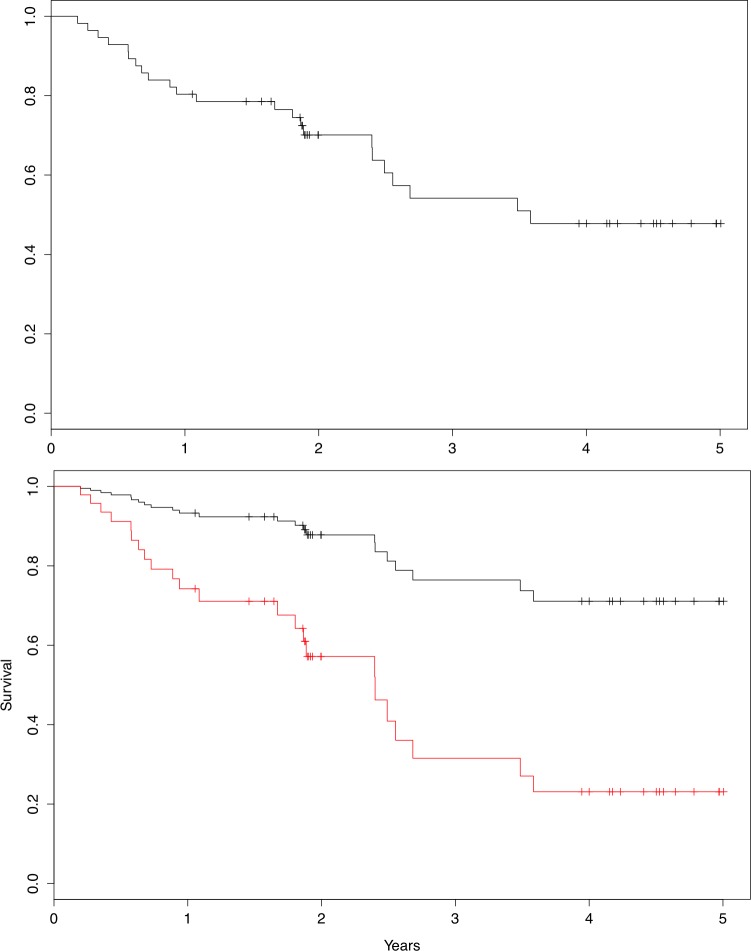
a) Kaplan-Meier plot 5-year over all-cause mortality. n = 56. b)Graphic cox regression for 5-year all-cause mortality shown by Peak 3h skewness below and above median. To adjust for established prognostic variables, including physical capacity (peak VO2), the models are adjusted for HFSS, which is held constant at its mean (8.45) between the two groups. Hazard Ratio = 1.44 per unit increase in skewness, p = 0.002.

60 patients were enrolled in the study over a period of 3 years. Four patients were excluded from the analysis due to a lack of accelerometer data. Demographic and clinical characteristics of the included subjects are presented in Table 1. The peak VO2 was on average 10.2 ml/kg (range 6.1–22.5 ml/kg). Follow-up time for surviving patients was 3 (range 1–5) years. Of 56 monitored patients, 23 died during follow-up (mean time 1.8 year, Fig 1). All mortality events were categorized as cardiovascular. The prognosis of each patient was estimated using peak VO2 and calculation of HFSS upon the patient’s enrollment in the study. The one-year survival risk estimate based on HFSS scores corresponded to 11% in 32 patients (corresponding to HFSS-score ≥ 8.10), 28% in 22 patients (corresponding to HFSS-score 7.20–8.09), and 40% in 2 patients (corresponding to HFSS-score ≤ 7.19) [27].

**Fig 2:**
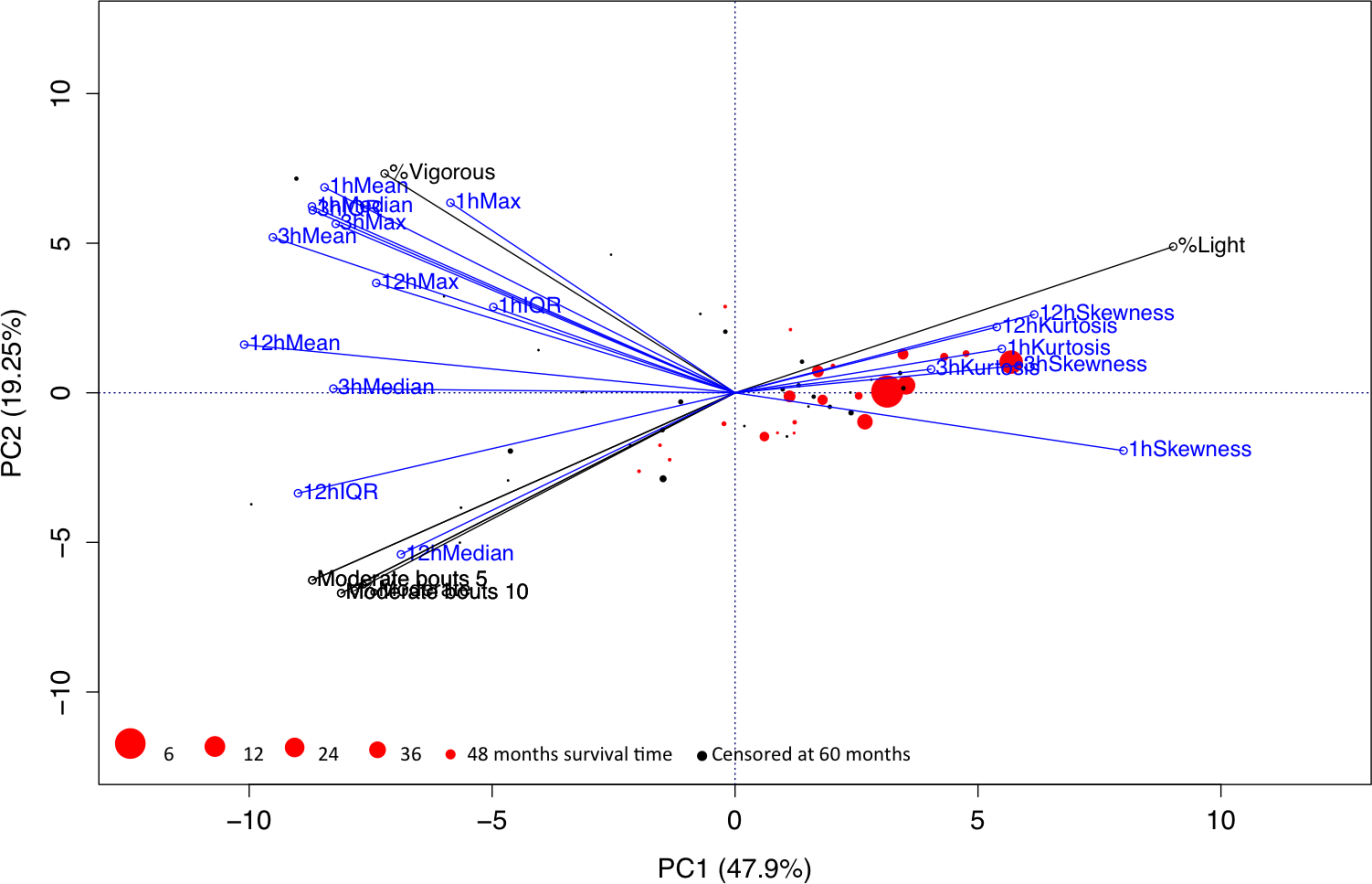
Principal components analysis (PCA) and biplot of all accelerometer derived variables. Variables measuring time spent physically active/inactive are colored black whereas novel variables based on periods with high physical activity are colored blue. Patient observations are colored black for censored data (survivors) and red for mortality events. The size of each observation corresponds to time until event, i.e. large circles correspond to early event and worse prognosis. Most of the total variance is captured with the two first principal components (69.15%) and the various variables thus covary to a large extent. The absolute majority of the observations with mortality events, in particular observations with early mortality events, are closely correlated with 1, 3 and 12 h skewness and 1, 3 and 12 h kurtosis.

**Fig 3:**
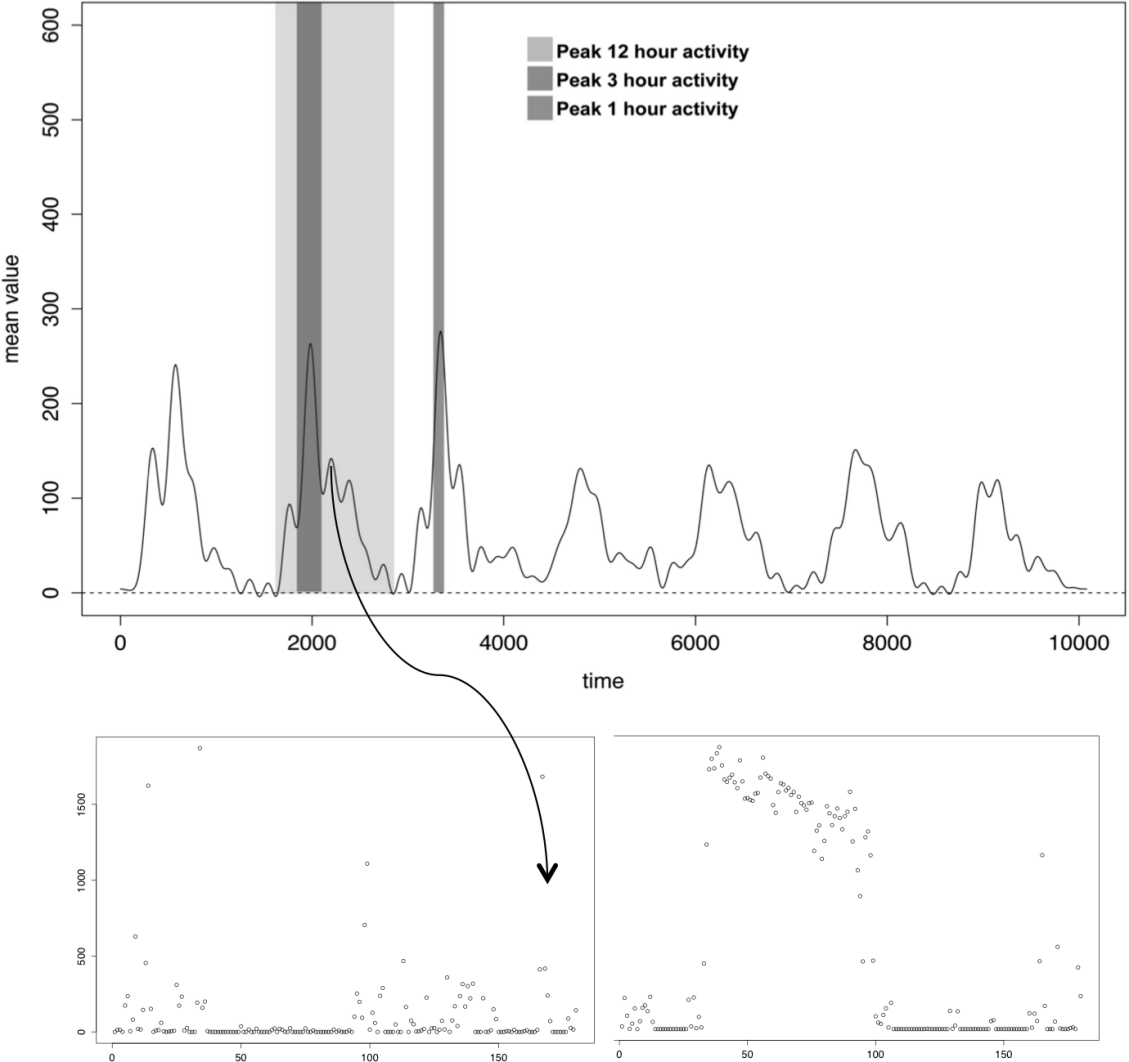
a) Raw data from a 7 day accelerometer recording with demarcations of peak 1, 3 and 12 hour periods identified using rolling mean. b)Peak 3h recording of physical activity identified by a rolling mean and characterized by high vs low degree of skewness.

All accelerometer-derived variables were analyzed for covariance using a principal components analysis (PCA) (Fig 2) and subsequently bi-plotted together with information on outcome in each individual observation. The PCA analysis revealed a high degree of covariance amongst the different accelerometer derived variables: 69.15% of variance was explained by principal component 1 and 2. The majority of patients (83%) encountering mortality events and 100% of patients with mortality events earlier than 36 months were above zero on PC1 and thus positively correlated with 1,3 and 12 h skewness and 1, 3 and 12 h kurtosis,Fig 3.

All accelerometer-derived variables were analyzed with regard to all-cause mortality and added to a baseline model utilizing HFSS scores. The accelerometer variables were subsequently ranked based on Bonferroni-corrected p-values (Table 2). The variables with the most significant contribution to mortality were 1, 3 and 12-h skewness, followed by various other measures of variability. Time spent being physically active, denoted as “time spent moderately active”, was associated significantly with mortality based on p-value, though not after correcting for multiple hypothesis tests. The HFSS score and skewness were not significantly correlated (r = −0.09; P = 0.50). The incremental predictive value of adding peak 3h skewness to a model with a HFSS risk score on the current patient cohort was tested. On its own, the score was associated significantly with the incidence of death (P<0.001; c-index 0.71; CI, 0.67–0.73). The addition of peak 3h skewness to the HFSS model significantly improved the predictive ability (likelihood ratio P<0.02), establishing skewness as a predictor of increased event rates when accounting for baseline risk. The c-index increased to 0.74 (CI, 0.69–0.78).

**Table 2:** 5-year all-cause mortality.

Variable	exp(coef)	se(coef)	p-value	Bonferroni-corrected
Peak1hSkewness	2.146	0.213	0.000	0.0115
Peak3hSkewness	1.444	0.108	0.001	0.0233
Peak12hSkewness	1.151	0.043	0.001	0.0387
Peak12hKurtosis	1.005	0.002	0.002	0.0713
Peak3hMean	0.996	0.001	0.002	0.080
Peak1hMedian	0.997	0.001	0.003	0.0913
Peak3hKurtosis	1.025	0.008	0.003	0.1010
Peak3hIQR	0.997	0.001	0.003	0.1097
Peak1hMean	0.998	0.001	0.004	0.1249
Peak12hMean	0.989	0.004	0.005	0.1838
Peak3hMedian	0.992	0.003	0.007	0.2292
Time spent at moderate physical activity	0.999	0.000	0.009	0.3209
Peak1hKurtosis	1.140	0.052	0.012	0.4167
Peak12hIQR	0.993	0.003	0.018	0.6184
Peak12hMax	1.000	0.000	0.030	1.0000
Peak3hMax	1.000	0.000	0.034	1.0000
Peak1hIQR	0.999	0.000	0.074	1.0000
Time spent at vigorous physical activity	0.968	0.018	0.077	1.0000
Peak1hMax	1.000	0.000	0.080	1.0000
Peak12hMedian	0.967	0.021	0.103	1.0000
Time spent at light physical activity	1.000	0.000	0.759	1.0000

5-year all-cause mortality Cox proportional hazards coefficients for the accelerometer-derived variables added to a baseline middle with HFSS. To correct for multiple hypothesis tests p-values are Boneferroni-corrected.

**Fig 4:**
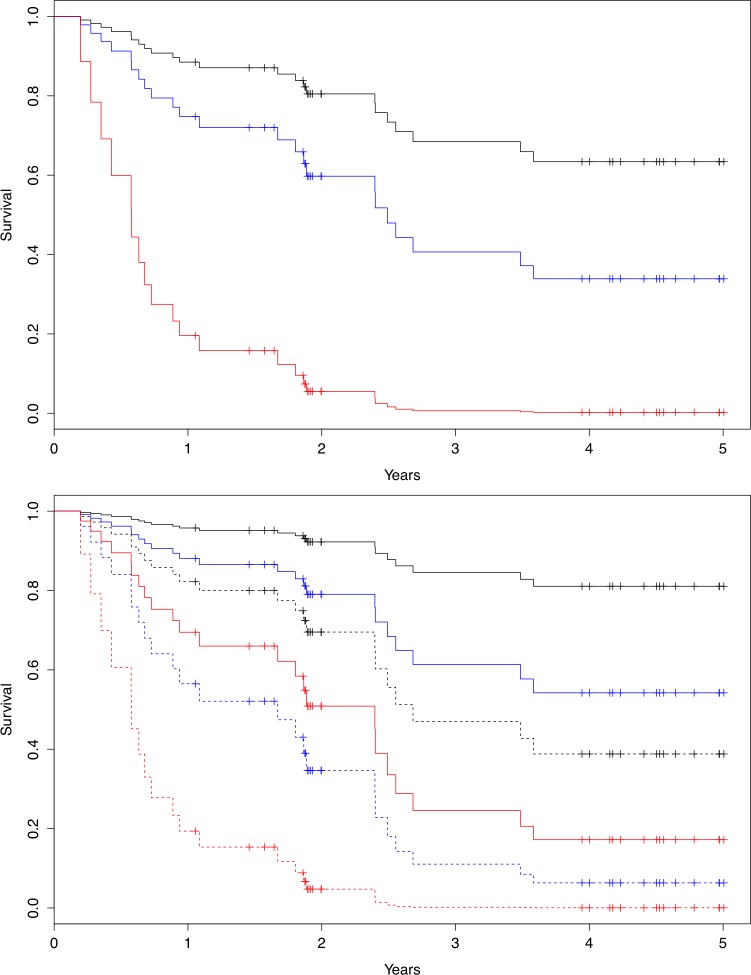
a) Graphic cox-regression over HFSS on 5-year all cause mortality, low risk (black), intermediate risk (blue) and high risk (red). b) Graphic cox regression on addition of Peak 3h skewness on top of HFSS-risk. Solid line denote skewness below median, dotted line denote skewness above median. Black denotes low risk, blue intermediate risk and red high risk based on calculated HFSS-score

Event incidence stratified patients into low-, medium-, and high-risk groups (Fig 4). The distribution of clinical characteristics of two equal sized groups based on peak skewness is summarized in Table 1. Continuous variables were tested using the t-test and frequencies were tested using the chi-square test. In summary, all clinical characteristics were distributed evenly between patients with high vs. low degrees of peak skewness.

## Discussion

The key finding of this study is that novel features derived from ambulatory PA assessed using an accelerometer can predict the outcome for patients with severe HF. Furthermore, these features provide additive prognostic information when added to an established risk model–HFSS.

Recent work has put forward not only maximum physical capacity but also the volume of PA in daily life as a relevant prognostic aspect in HF [20,21,28]. Overall, these studies demonstrate that physically active patients have a better prognosis than patients who are not physically active, and this also is correlated to the physical capacity of the patient [20]. An intriguing observation is the linear correlation between time spent being highly physically active and physical capacity/peak VO2 in HF patients [29]. This questions whether such information has an additive prognostic value in patients whose physical capacity is already known from assessment with peak VO2 CPX [22,29]. Our data, confirms that time spent being physically active is significantly associated with the prognosis in HF and that it can be assessed by means of a waist-worn accelerometer (see Table 1). When added to peak VO2 or HFSS, “time spent moderately active” remained significantly associated with mortality (p = 0.009), but after correcting for multiple hypothesis tests, none of the variables based on time spent being physically active remained significant. Thus, the time spent being physically active co-varies to a large degree with physical capacity in heart failure, which undermines its additive value in patients whose physical capacity is known.

In the present study we aimed therefore to investigate whether other more qualitative or descriptive aspects of PA, rather than mere volumes of activity at different intensities, could be used to establish a prognosis for patients with severe HF. Step length is in fact a variable that distinguishes patient with severe HF from those suffering from milder forms of HF [23]. Thus in the current study the underlying idea was that patients with a worse prognosis would exhibit a walking pattern characterized by both a low walking speed and frequent pauses. We hypothesized that this would lead to high variance in the physical activity recorded by the accelerometer. Skewness and kurtosis were used to assess walking pattern as they are measures of how physical-activity intensity is distributed over time. The strategy proved successful with regard to prediction of patient outcome: In accordance with our hypothesis, walking patterns recognized through analysis of skewness were highly correlated with outcome. Second to skewness, 12 hour kurtosis was the variable most significantly related to all-cause mortality, albeit not after Bonferroni-correction.

Significance of skewness was tested by adding it to established prognostic markers in accordance with the recommendations of the AHA on the evaluation of potential new prognostic markers in HF [8]. Peak VO2 is probably the single factor with highest predictive value with regard to mortality in HF; its accuracy is refined further when combined with clinical variables in HFSS [9,11,13,30]. A feature that distinguishes HFSS and peak VO2 from other prognostic models in HF is that both peak VO2 alone and HFSS (by the inclusion of peak VO2 in the model) take the patient’s physical performance into account when determining a rough prognosis. This makes them well suited as baseline models when searching for new prognostic factors based on PA, since the addition of a variable that merely reflects exercise performance would provide little or no prognostic variance on top of peak VO2 or HFSS. The addition of for example peak 3h skewness to the HFSS model increased the c-index from 0.71 to 0.75, and the model differed significantly from the baseline model likelihood ratio test (p = 0.002). This illustrates not only that accelerometer derived skewness is a promising new prognostic marker in heart failure, but also that it measures an aspect of PA that is not merely a consequence of the patient’s physical capacity.

An important aspect of the current study, and one that strengthens its results in comparison to other reports, is that the study population had a consistently low (NYHA III-IV) functional class rather than a wide spectrum of functional capacities. When comparing patients in different functional classes, there is an inherent risk that correlations are powered by large differences between the groups rather than by accurate discrimination of patients with a similar functional status: it comes as no surprise that in a comparison of asymptomatic patients with functionally severely impaired patients, there is a substantial difference in several aspects of PA. By contrast, accurately distinguishing patients in a homogenous population based on their prognosis poses a real challenge. The robust nature of the correlations between peak 3h skewness and mortality in the clinically homogenous population of the present study strengthens the relevance of the findings, especially since establishing a prognosis for this category of patient is quite difficult. In fact, based on peak VO2, most of the patients were in the prognostic “intermediate range”, in which the accuracy of currently established prognostic tools is much debated [31]. Still, this is a single-center study in which patients who satisfied the inclusion criteria were asked to participate in conjunction with their admittance for CPX and peak VO2 measurements as part of the clinical routine over the course of several years with no recorded instances of patients declining to participate or of drop-outs. Therefore, the study has limitations with regard to its validity for the general HF patient population, both over a wide range of disease severity and in different healthcare settings. The small number of observations also precludes a search for a more pervasive model including additional accelerometer-derived variables, but based on the apparent additive value of peak skewness on the predictive accuracy of peak VO2 and HFSS it strongly supports the addition of accelerometer-derived variables to improve the accuracy of these prognostic models even further.

This is the first time analysis of variability in physical activity has been investigated as a prognostic marker in HF and therefore the results must be regarded as preliminary until validated. Since prognostication using estimation of peak VO2 is both tedious, challenging for the patient and the method is generally available only at tertiary centers, the prospect of a tool to estimate prognosis that involves only minimal inconvenience for the patient while at the same time easy to manage and relatively low-cost would be close to ideal. Albeit speculative, based on its ease of use and risk-free non-invasive nature, addition of accelerometers to the clinical assessment of HF patients would require relatively little effort and thus possibly reduce the known under-utilization of available therapies in elderly, frail patients with multiple co-morbidities [32]. The results of the present study indicate that such a measurement could be added to established prognostic markers and thus improve the prognostic accuracy with regard to mortality. This could have an important impact on clinical decision-making, in particular on transplantation and assist-devices (LVAD) since these decisions are based mainly on estimated short-term mortality.

## Conclusion

In conclusion, walking pattern can be identified and characterized through analysis of data from accelerometers, and this variable has an additive value over and above physical capacity in a prognostic model involving patients with HF. The findings from the present study emphasize the use of accelerometer analysis in clinical practice both to extract features of PA relevant to functional classification and to make more accurate prognoses.

## Supporting Information

S1 File Supporting Information File. (XLSB)
